# Complete Inversion of the Bladder, and the Protrusion of the Organ at the Urethra

**Published:** 1872-01

**Authors:** 


### MISCELLANEOUS.

Complete Inversion of the Bladder, and the Protrusion of the Organ at the Urethra.—In the "Transactions of the Wisconsin State Medical Society," as reported in the Northwestern Medical and Surgical Journal, we find the report, by Dr. S. A. Ferrin, of Mortford, of a case of complete protrusion of the bladder through the urethra. The accident occurred in a lady during a protracted labor. Dr. Ferrin first saw the case thirty-six hours after labor commenced, and at the upper part of the vulva, between the pudendal lips, found a tumor about the size of a small hen's egg, pyriform in shape, the base being below and the apex above. Finding it did not come from the vagina, he delivered the lady, with forceps, of a large, healthy child, weighing twelve pounds. After waiting an hour for the body to react, he made a further examination, and found the tumor somewhat smaller, rough and granulated, of a bright red or florid color, somewhat hard and unyielding to pressure. He could not discover the orifices of the ureter. No pain on handling. There was a constant dribbling of urine, which had commenced with the appearance of the tumor, thirty hours before the delivery, as he was informed by the physician in attendance. The reduction was effected by means of a common silver female catheter applied to the fundus of the tumor, and carrying it up in the direction of the urethra. After the reduction, the urethra was so dilated that it readily admitted the middle finger its entire length. The hips were elevated, a T bandage applied, and perfect rest enjoined. The catheter had to be used for three or four days; otherwise the patient made a good recovery.

